# Screening, Identification and Physiological Characteristics of *Lactobacillus rhamnosus* M3 (1) against Intestinal Inflammation

**DOI:** 10.3390/foods12081628

**Published:** 2023-04-12

**Authors:** Jiayan Jiang, Ke Li, Yuanliang Wang, Zhongqin Wu, Huiqin Ma, Shilin Zheng, Zongjun Li

**Affiliations:** College of Food Science and Technology, Hunan Agricultural University, Changsha 410128, China

**Keywords:** lactic acid bacteria, probiotics, antibacterial, safety evaluation, flavor, screening

## Abstract

The probiotic role of lactic acid bacteria (LAB) in regulating intestinal microbiota to promote human health has been widely reported. However, the types and quantities of probiotics used in practice are still limited. Therefore, isolating and screening LAB with potential probiotic functions from various habitats has become a hot topic. In this study, 104 strains of LAB were isolated from and identified in traditionally fermented vegetables, fresh milk, healthy infant feces, and other environments. The antibacterial properties—resistance to acid, bile salts, and digestive enzymes—and adhesion ability of the strains were determined, and the biological safety of LAB with better performance was studied. Three LAB with good comprehensive performance were obtained. These bacteria had broad-spectrum antibacterial properties and good acid resistance and adhesion ability. They exhibited some tolerance to pig bile salt, pepsin, and trypsin and showed no hemolysis. They were sensitive to the selected antibiotics, which met the required characteristics and safety evaluation criteria for probiotics. An in vitro fermentation experiment and milk fermentation performance test of *Lactobacillus rhamnosus* (*L. rhamnosus*) M3 (1) were carried out to study its effect on the intestinal flora and fermentation performance in patients with inflammatory bowel disease (IBD). Studies have shown that this strain can effectively inhibit the growth of harmful microorganisms and produce a classic, pleasant flavor. It has probiotic potential and is expected to be used as a microecological agent to regulate intestinal flora and promote intestinal health. It can also be used as an auxiliary starter to enhance the probiotic value of fermented milk.

## 1. Introduction

Changes in the structure and quantity of intestinal microflora greatly impact human health and the occurrence of diseases. The gut microbiome is known as the ‘invisible endocrine organ’, the place where the human body digests food and absorbs nutrients. LAB, as one of the intestinal bacteria, are widely distributed and can be isolated from the environment, food, and human and animal gastrointestinal tracts [1]. Among them, strains with probiotic properties have high resistance to acid and bile salts, can improve immunity, and have antibacterial, antioxidant, and free radical scavenging activities. They can improve gastrointestinal function, regulate intestinal flora, and promote the apoptosis of human cancer cells [2,3]. Probiotics are defined as living microorganisms that can bring health benefits to the host when taken in sufficient quantities [4]. With the continuous discovery and exploration of the probiotic properties of LAB, many strains have been proven effective and safe, and have been developed for general use.

IBD is an inflammatory state of the colon and small intestine caused by an immune response disorder. It is one of the common gastrointestinal diseases, which include Crohn’s disease and ulcerative colitis, usually accompanied by diarrhea, pain, fatigue, constipation, and other symptoms. Epidemiological studies have shown that the incidence of IBD is on the rise worldwide and may greatly impact human health and life. However, the exact cause of the disease is still unclear. Factors such as susceptibility genes, the immune system, and external environmental stimuli may lead to the occurrence of IBD [5]. Recently, the imbalance in intestinal flora has become an IBD research hotspot; microbial imbalance has been shown to be closely associated with allergy, eczema, IBD, and irritable bowel syndrome [6]. Harmful microorganisms not only affect food quality and safety but also, to some extent, threaten the life and health of the host. Antibiotics are commonly used in clinical practice to prevent and treat bacterial infections. However, because of the improper use of antibiotics, bacterial drug resistance has been gradually increasing, compounding the difficulty of controlling bacterial infections. Probiotics have been shown to inhibit the growth and colonization of pathogenic bacteria by inducing the host to produce organic acids, bacteriocins, and other substances; alter the intestinal microbial community; and improve the body’s intestinal function. To strengthen the intestinal barrier by maintaining tight junctions and inducing mucin production, mediated immune regulation may mediate the secretion of cytokines and immunoglobulins through signaling pathways such as Nuclear Factor kappa-B, affecting the proliferation and differentiation of immune cells or epithelial cells. LAB play a crucial role in regulating the composition and metabolism of intestinal microflora and intestinal immune function. This makes them beneficial in preventing and treating IBD and other diseases [6,7]. LAB with antibacterial ability can be used as probiotics to partly replace antibiotics, avoid bacterial resistance, and improve human and animal health [8]. However, probiotics’ production and survival in the human gastrointestinal tract are complex processes. Probiotics should survive in the presence of gastric acid and bile, and have the ability to colonize and play a role in the intestine [9]. Therefore, being nontoxic, harmless, nonpathogenic, acid and bile resistant, and able to proliferate in the presence of bile are the essential criteria for potential probiotic strains. Therefore, to screen out potential probiotics, it is a necessity and a priority to identify and evaluate their physiological characteristics and safety. Previous studies have shown that naturally fermented foods and dairy products are important sources of probiotic strains. In addition, probiotics for human use are best sourced from humans because such microorganisms may be more competitive and better adapted to human microenvironment conditions than probiotic strains from other sources. In this study, LAB with probiotic potential were isolated and identified from traditionally fermented vegetables, fresh milk, healthy infant feces, and other habitats. The inhibition of pathogenic bacteria, acid and bile salt resistance, adhesion ability, safety, intestinal flora regulation ability, and fermentation performance were evaluated in vitro. The aim was to obtain new probiotic resources and lay a foundation for subsequent research and application.

## 2. Materials and Methods

### 2.1. Sample Collection

The sources of fermented vegetables were 3 fermented bamboo shoots from Yiyang, Hunan Province, and 9 fermented vegetables sold in Tongdao County, Huaihua City, Hunan Province. Three feces samples we obtained from obese people and three from infants and children (0–3 years old), collected from schools. Raw milk sources produced 3 samples from the Hunan Institute of Animal Husbandry.

### 2.2. Reagents

The reagents and materials used are shown in Table 1:

### 2.3. Isolation and Purification of Bacterial Strains

On an ultra-clean bench, 25 g or 25 mL of the sample was placed in 225 mL of sterile saline containing glass beads and shaken well in a constant temperature shaker at 37 °C. The sample solution was diluted gradually, and 100 μL of the appropriate diluent was coated on an MRS agar plate and anaerobically cultured at 37 °C for 24–48 h. Single colonies were picked from countable MRS agar plates and repeatedly streaked on MRS agar medium to further purify the colonies. The purified bacteria were transferred to MRS broth at 37 °C for 12 h, mixed with 50% glycerol by volume at 7:3, and stored at −20 °C. Colonies were scribed in MRS agar medium slant, cultured at 37 °C until the growth of colonies was detected, preserved at 4 °C for identification, and placed in glycerol preservation at −80 °C. 

### 2.4. Identification of LAB

Strain identification generally clarifies the taxonomic relationship between microbial populations through morphological characteristics, physical and chemical properties, genetic evolution, and other aspects used to confirm the identity of strains. The physiological and biochemical characteristics of the screened strains were detected according to the ‘Manual for Systematic Identification of Common Bacteria’ (eighth edition) and ‘Berger’s Manual of Bacteria’ [10]. DNA was extracted from Gram-positive and catalase-negative strains using a bacterial genomic DNA extraction kit. The 16 S rDNA gene was amplified, and the universal primers of bacteria were selected for amplification primers: the forward primer 27F: 5′-AGAGTTTGATCCTGGCTCAG-3′ and reverse primer 1492R: 5′-AAGGAGGTGATCCAGCC-3′. The PCR reaction mixture was prepared. After the amplification reaction, the PCR product was separated by electrophoresis, stained, and checked on the ultraviolet illuminator. The clear target band was selected and sent to Sangon Biotech (Shanghai, China) Co., Ltd., for gene sequencing. The obtained sequence was subjected to the BLAST program for sequence homology alignment. BioEdit sequence alignment was used to edit forward and reverse sequences, and the isolates were identified according to the sequence. Phylogenetic trees were constructed using Mega-x software. 

### 2.5. Antibacterial Experiment

The Oxford cup method [11] was used to screen LAB with active antibacterial substances, and common pathogenic bacteria in food were selected as indicator bacteria. Selected indicator bacteria were *S. aureus* ATCC 6538, *L. monocytogenes* ATCC 19115, *E. coli* CGMCC 9181, *Pseudomonas aeruginosa*, and *Bacillus subtilis*. The indicator bacteria were activated in LB broth for three generations, cultured at 37 °C for 24–36 h; 10^6^ CFU/mL was taken as the concentration of indicator bacteria suspension, and the indicator bacteria were stored in a refrigerator. The screened LAB were activated in an MRS broth medium for three generations and then inoculated in MRS broth with 1% inoculation. After anaerobic culture at 37 °C for 48 h and 8 min centrifugation at 10,000 r/min, the fermentation supernatant was taken to obtain the crude extract of LAB. A total of 200 μL indicator bacteria was coated on the LB agar medium, and the sterile Oxford cup was buckled on the LB agar medium with a specific strength. Then, 200 μL crude extract of LAB was added to the Oxford cup, and standard MRS was performed as a blank control. Three experiments were repeated for each LAB. The plate was transferred to a constant temperature and humidity incubator and cultured at 37 °C. After 24 h, the diameter of the inhibition zone was measured accurately with a vernier caliper wherever the inhibition zone was observed.

### 2.6. Determination of LAB Tolerance

#### 2.6.1. Acids Resistance Test

The activated LAB were cultured in MRS liquid medium in a 37 °C anaerobic environment for 24 h, centrifuged at 4 °C, 10,000× *g* for 8 min, and washed twice with sterile PBS buffer (pH 7.2) or 0.9% sterile saline. The bacteria were resuspended in sterile MRS broth medium at pH 2.0 and 3.0 and incubated anaerobically at 37 °C for 3 h. Samples were taken at 0 h and 3 h for dilution, respectively, and 100 μL of the appropriate gradient dilution was spread and inoculated on MRS agar medium and incubated under anaerobic conditions for 24 to 48 h. The standard MRS broth in which the cultures were inoculated was used as a positive control [12]. To calculate its survival rate, we used the formula:

Survival rate (%) = Log*N_t_* (CFU)/Log*N*_0_ (CFU) × 100%

where *N_t_* is the number of viable bacteria after treatment and *N*_0_ is the number of added live bacteria. 

#### 2.6.2. Bile Salt Resistance Experiment

In order to detect the LAB tolerance of bile in a low pH environment (growth and survival at pH 2.0), MRS broth medium with 0.2% and 0.3% pig bile salt concentration was prepared and sterilized using a 0.22 μm filter membrane. The activated bacterial liquid was mixed and centrifuged at 4 °C, 10,000× *g* for 8 min, and the organisms were washed twice with saline buffer. Bacteria were resuspended in different concentrations of bile salt medium, and positive control groups were established. Samples were taken at 0 h and 3 h after incubation for gradient dilution, and 100 μL of the appropriate gradient dilutions was inoculated on MRS agar medium. The survival rate was calculated using the formula described for acid tolerance assay [13]. 

#### 2.6.3. Simulated Gastrointestinal Fluid Tolerance Experiment

Probiotics need to tolerate not only lower pH and bile salts but also pepsin and trypsin in the gastrointestinal fluid to colonize and function in the human body. The artificial gastric and intestinal fluids were prepared with reference to the literature [14,15]. To simulate gastric fluid, hydrochloric acid was diluted to adjust the pH to 2.0 and 3.0, 10 g of pepsin was added, and 1000 mL of water was used to fix the volume. To simulate intestinal fluid, 6.8 g of potassium dihydrogen phosphate solution dissolved in water was used, and the pH was adjusted to 6.8 with NaOH solution. A total of 10 g of pancreatin was dissolved in water, and 1000 mL of water was added to fix the volume. The simulated gastric and intestinal fluids were filtered with a 0.22 μm filter membrane for sterilization and set aside.

The bacterial suspension was inoculated into the simulated gastric fluid, incubated anaerobically at 37 °C, and the survival rate was calculated by sampling and counting at 0 h and 3 h. At the same time, 1 mL of simulated gastric fluid containing bacteria was added into 9 mL of simulated intestinal fluid and incubated anaerobically at 37 °C, and the survival rate was calculated by sampling and counting at 0 h and 3 h in the simulated intestinal fluid. The formula for calculating the survival rate was described in the acid resistance test. 

### 2.7. Determination of Adhesion Ability of LAB

The complete medium (fetal bovine serum: Burke modified Eagle high-glucose medium = 1:9) was prepared. The Caco-2 cells preserved in liquid nitrogen were quickly thawed in a 37 °C water bath, transferred into a cell culture flask containing a complete medium, blown evenly, and cultured in a 5% CO_2_ incubator at 37 °C. The medium was changed according to the cell growth. When the cell fusion degree reached 80–90%, the cells were subcultured and digested with 0.25% trypsin solution. The cells were used in the experiment after 3 passages. The cells were resuspended in a complete medium to a 5 × 10^4^/mL concentration and inoculated into 96-well plates. The cells were cultured until the cell monolayer covered the plate holes.

The weighted bacteria with a concentration of 1 × 10^8^ CFU/mL was suspended in a complete medium containing 10% serum. The medium in the perforated plate was sucked, the sterile PBS buffer preheated at 37 °C was slowly washed twice, 200 μL of LAB suspension was added to co-culture with cells, and a cell-free blank group was prepared. All were incubated in a carbon dioxide incubator for 4 h. After incubation, the suspension containing the cell pores was removed and washed with preheated sterile PBS buffer 3 times, and the non-adherent bacteria were washed away. The cells were digested with 0.25% trypsin, digested, and mixed. A total of 100 μL of the mixture (the suspension was taken directly from the blank group) was selected and spread on MRS agar medium for culture. The adhesion rate was counted and calculated with the formula described in the acid resistance test.

### 2.8. In Vitro Safety Experiment

#### 2.8.1. Hemolytic Test

Hemolysis is the rupture of erythrocytes and escape of hemoglobin under the action of hemolytic toxins and other physicochemical factors, and the grades of hemolysis can be divided into: complete hemolysis (β hemolysis), partial hemolysis (α hemolysis), and no hemolysis (γ hemolysis) [16]. The screened strains were scribed on Columbia blood agar plates and incubated anaerobically at 37 °C for 24 h. The colonies and blood agar plate morphology were observed, and *S. aureus* was used as a positive control.

#### 2.8.2. Antibiotic Sensitivity Test

The antibiotic susceptibility of the screened strains was assessed by the paper diffusion method, and the susceptibility of 13 antibiotics, including Clindamycin, Streptomycin, Gentamicin, Kanamycin, Vancomycin, Tetracycline, Chloramphenicol, Ampicillin, Metronidazole, Bacitracin, Cotrimoxazole, Neomycin, and Ofloxacin, was tested by the paper diffusion method [17]. The LAB solution of 200 μL MRS broth was cultured for 24 h, evenly coated on MRS agar medium, and left to wait for the plate to dry. Sterile antibiotic paper sheets were attached to the plate with the center of each paper sheet greater than 24 mm apart and the paper sheets greater than 15 mm from the inner edge of the plate. Three to four paper sheets were attached to each dish placed at 4 °C for 30 min for antibiotic diffusion and then incubated anaerobically at 37 °C for 24 to 48 h. The diameter of the inhibition zone was measured precisely with vernier calipers, and the criteria for determining drug resistance were taken from the Executive Standard for Antimicrobial Drug Susceptibility Testing [18], shown in Table 2.

### 2.9. In Vitro Fermentation and Detection

#### 2.9.1. In Vitro Fermentation

1. Sample collection: This study was approved by the Biomedical Research Ethics Committee of the Hunan Agricultural University (Protocol Code: Ethical Review 2022 No. 117, 10 November 2022). All participants provided written informed consent. Fecal samples were collected from 8 donors with diarrhea symptoms, all of whom had no underlying diseases and had not taken antibiotics within 3 months before collection. The collected samples were mixed with the same amount of feces and frozen in a refrigerator at −80 °C until use;
2. Preparation of basal medium [19]: The medium composition (/L) was yeast extract 2.0 g, peptone 2.0 g, sodium chloride 0.1 g, potassium dihydrogen phosphate 0.04 g, sodium bicarbonate 2.0 g, calcium chloride 0.01 g, magnesium sulfate 0.01 g, hemin 50 mg, vitamin K 10 μL, bile salt 0.5 g, L-cysteine 0.5 g, and resazurin 1.0 mg. The basic medium was divided into test tubes and sterilized at 121 °C for 20 min. After sterilization, the medium was removed and placed in an anaerobic incubator for 18–24 h to ensure the container was anaerobic before the experiment. The expected color of the basic medium in the anaerobic environment was light yellow, and the medium was to turn red rapidly in the presence of oxygen;
3. In vitro fermentation: The feces were taken out and mixed evenly in a sterile container, and sterile normal saline with a weight of 9 times the weight of the feces was added to mix thoroughly. The gauze was filtered to obtain a 10% fully mixed fecal suspension. Then, 10% fecal suspension was added to each test tube. The experimental group received 10% LAB suspension with a concentration of about 10^8^ CFU/mL, and the same amount of sterile water was added to the control group. The samples were cultured in an anaerobic incubator at 37 °C for 24 h, and the samples were collected aseptically at 0 and 24 h, respectively. The samples were taken out and immediately placed in ice water to stop fermentation and then stored at −80 °C.

#### 2.9.2. DNA Extraction and Sequencing

Three groups of parallel samples of each bacteria were mixed evenly, and the genomic DNA of the samples was extracted by the CTAB method. The purity and concentration of total DNA were detected by 0.8% agarose gel electrophoresis. PCR amplification was performed using specific primers with Barcode and high-fidelity DNA polymerase, forward primer 515F (5′-GTGCCAGCMGCCGCGGTAA-3′), and reverse primer 806R (5′-GGACTACHVGGGTWTCTAAT-3′). Each sample was repeated 3 times to ensure all PCR reactions terminated in the linear amplification period. After PCR, the PCR products of the same sample were mixed and detected by 2% agarose gel electrophoresis. The detection condition was 5 V/cm and the detection time was 20 min. The target fragment was cut and recovered. The target DNA fragments were recovered by elution with TE buffer, and the PCR amplification products were quantified by the QuantiFluor TM-ST blue fluorescence quantitative system. The library was constructed using the NEB Next^®^ Ultra TM DNA Library Prep Kit according to the mixing ratio, and the quality and sequencing were performed by Agilent Bioanalyzer 2100 and Qubit. The double-ended sequencing results were spliced by FLASH, and the quality control screening was performed to obtain high-quality target sequences. Subsequent bioinformatics operations were completed using QIIME, Usearch, etc., and statistics and mapping were mainly completed through R. 

#### 2.9.3. Detection of PH Value and Determination of Short-Chain Fatty Acids (SCFAs)

The pH values of the fermentation culture at 0 h and 24 h were measured using a handheld pH meter. Each sample was measured 3 times, and the mean and standard deviation were calculated by repeated measurements. 

The content of SCFA was determined by gas chromatography (GC), referring to a reported method [20], with some modifications. The fermentation broth was mixed with 25% metaphosphoric acid solution with a 4:1 ratio, vortexed for 1 min, centrifuged at 8000 r/min for 4 min, and filtered by 0.22 μm organic microporous membrane. DB-FFAP gas chromatographic column (30 m × 250 μm × 5 μm) was used. The carrier gas was N_2_, and the flow rate was 0.8 mL/min. The auxiliary gas was H_2_. The FID detector temperature was 270 °C, the injection port temperature was 250 °C, the split ratio was 5:1, and the injection volume was 1 μL at 220 °C. Mass spectrometry (MS) conditions were: voltage of 70 eV, ion source temperature of 230 °C, mass scanning range of *m*/*z* 28–300, and electron ionization (EI) mode. The mass spectra of the compounds were compared with the NIST 20. L mass spectrometry database, and compounds with a matching degree greater than 80% were extracted for analysis.

### 2.10. Study on the Milk Fermentation Performance

LAB with better comprehensive performance were selected for milk fermentation, and their pH and volatile flavor substances were measured and analyzed to explore the fermentation performance of the screened LAB.

#### 2.10.1. Preparation of Fermented Milk 

Activation of bacteria: After anaerobic culture at 37 °C for 24 h, the activated bacterial solution was mixed and centrifuged at 4 °C and 10,000× *g* for 8 min, then washed twice in the saline buffer. The collected cells were resuspended in saline to prepare a bacterial suspension, and the concentration of LAB reached about 10^9^ CFU/mL. 

Preparation of fermented milk: Whole milk powder was added to distilled water at 50 °C, heated to 60 °C, stirred for 15 min, then placed in a water bath at 50 °C for 30 min. After hydration, the sample was heated again to 65 °C, 20 MPa homogenization, sterilized at 95 °C for 5 min, then immediately cooled in an ice water bath, to about 37 °C and inoculated with 1% inoculum. The fermentation was completed at 4 °C after 12 h. 

#### 2.10.2. PH Changes

For PH changes, 150 mL of pure milk was added to a 300 mL conical flask. After pasteurization, milk was cooled, inoculated with 1% inoculation amount, and fermented at 37 °C. From the start of the inoculation, the pH value of fermented milk was measured every 2 h using a handheld pH meter. 

#### 2.10.3. Determination and Analysis of Volatile Flavor Compounds

In sample pretreatment, 6 mL of fermented milk was added to the sample bottle, and the extraction head was aged at 250 °C for 5 min at the inlet, then vertically inserted into the sample bottle to extract the flavor compounds of fermented milk. The extraction conditions were 60 °C temperature, 300 r/min rotation speed of magnetic stirrer, and 50 min extraction time [21]. 

The extraction head was inserted vertically into the forward sample port on the machine and desorbed for 5 min. The initial gas chromatography conditions were a temperature of 35 °C and a time duration 5 min; then, the temperature was increased to 140 °C at a rate of 5 °C/min and maintained for 2 min. It was raised again to 10 °C/min 250 °C. The carrier gas was high-purity helium (He), the carrier gas flow rate was 1.0 mL/min, and the injection was not split. Mass spectrometry conditions were electron energy 70 eV, ion source temperature 230 °C, ionization mode EI, and mass scanning range *m*/*z* 35–500. The emission current was 100 μA, and the detection voltage was 1.4 k V. Qualitative and comparative analyses were conducted, and data were exported for CAS number query.

### 2.11. Statistical Analysis 

All tests were conducted in triplicate, and the results are shown as mean ± standard deviation. SPSS statistical analysis software (IBM SPSS Statistics 23) was used for analysis. The data were evaluated by one-way ANOVA, analyzed, and plotted using Origin 2018, and Duncan’s multiple range test was used to analyze the significant differences.

## 3. Results and Discussion

### 3.1. Analysis of Basic Physiological Characteristics of LAB Strains

**Figure 1:**
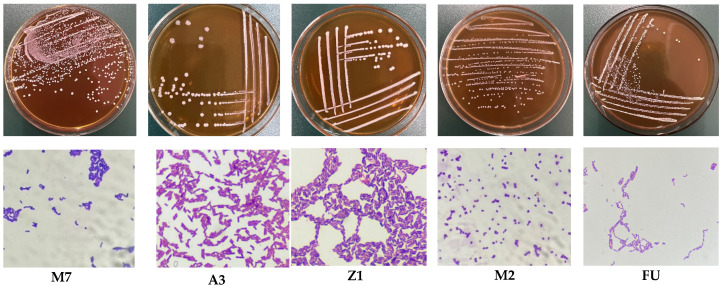
Some bacterial colony morphology similar to LAB, through microscopic examination. The selected bacteria were Gram-positive, hydrogen-peroxide-negative, and non-motile bacteria. M7 and FU were single-rod-shaped, A3 and Z1 were rod-shaped, and M2 was cocci.

A total of 228 strains of bacteria were isolated and purified from traditionally fermented vegetables, 108 strains of bacteria were obtained from feces, and 68 strains of bacteria were obtained from raw cow’s milk. After morphological observation, Gram staining, microscopic examination, and the identification of physiological and biochemical characteristics, we obtained 134 strains of bacteria that conformed to the basic physiological characteristics of LAB. The colony morphology of these strains was round and shiny, with a central protrusion and flush edges, creamy white or off-white in color, 0.5–1.5 mm in diameter, Gram-positive, and all negative for hydrogen peroxide contact enzyme, and their microscopic examination showed a single pair of rods or a spherical structure with no movement and no spores (Figure 1). 

### 3.2. Molecular Identification Results and Analysis

**Figure 2:**
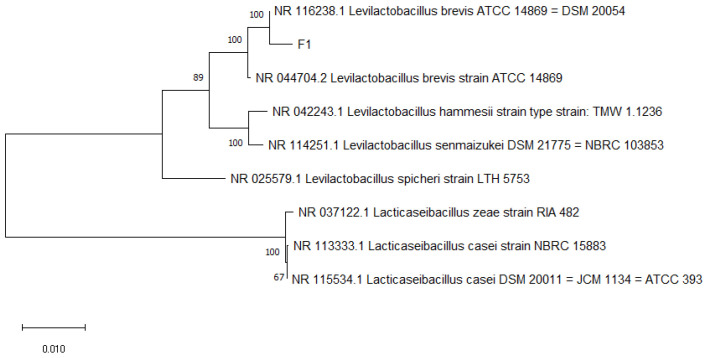
F1 gene development tree.

The genome extraction, PCR amplification, and sequencing of 134 strains of suspected LAB were carried out, and the gene sequences of the strains were compared using BLAST, and phylogenetic tree was established (Figure 2). The results indicated that 103 strains of LAB were obtained in this screening, and some results are indicated in Table 3.

Among these strains, 53 LAB strains were isolated from traditionally fermented vegetables and screened, including 22 strains of *Lactobacillus plantarum* (*L. plantarum*) and 31 strains of *Lactobacillus brevis* (*L. brevis*). The other obtained LAB were relatively small or single in number. This may be related to the composition of nutrients in traditionally fermented vegetables and the lower pH environment and because *L. plantarum* and *L. brevis* may be the dominant strains in the screened fermented vegetables. Twenty-nine strains of LAB were isolated from feces samples, including fifteen strains of *Enterococci*, eight strains of *Lactobacillus paracasei* (*L. paracasei*), two strains of *L. brevis*, one strain of *Bifidobacterium crudilactis* (*B. crudilactis*), one strain of *L. plantarum*, one strain of *Pediococcus pentosaceus* (*P. pentosaceus*), one strain of *Westerella sibiricus*, and one strain of *Streptococcus salivarius* (*S. salivarius*). Twenty-one strains of LAB were isolated from raw milk, including two strains of *L. rhamnosus*, six strains of *L. paracasei*, two strains of *Bifidobacterium thermophilus*, four strains of *B. crudilactis*, two strains of *P. pentosaceus*, one strain of *Lactobacillus parabuchneri* (*L. parabuchneri*), one strain of *Lactobacillus buchneri*, and two strains of *Enterococcus faecium* (*E. faecium*).

### 3.3. Antibacterial Experiment Results and Analysis

Probiotics can secrete organic acids, polysaccharides, antimicrobial peptides, and other substances to inhibit the growth of other microorganisms [22]. In this experiment, the representatives of *Gram-positive bacteria*: *S. aureus*, *Listeria*, and *Bacillus subtilis*, and *Gram-negative bacteria*: *Pseudomonas aeruginosa* and *E. coli* were used as indicator bacteria to detect the antibacterial activity of the screened LAB. A total of 101 strains were excluded because of repetitive LAB; 72 strains of LAB were subjected to the bacteriostatic test, excluding LAB without a bacteriostatic ring and a smaller bacteriostatic ring, leaving 16 strains of LAB with a better comprehensive bacteriostatic level.

**Figure 3:**
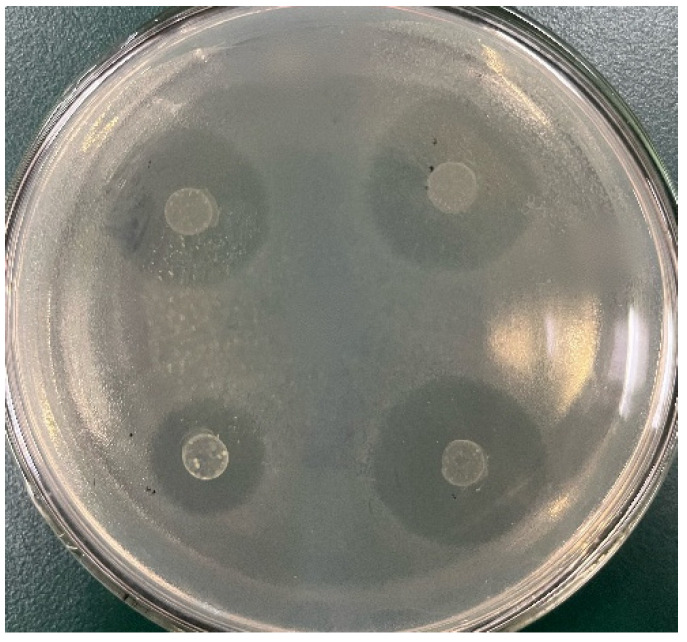
Antibacterial effect of M16 on *S. aureus*.

The diameter of the inhibition zone is shown in Table 4, and the morphology of Antibacterial circle is shown in Figure 3.The crude extracts of 16 strains of LAB showed certain antibacterial effects. They have good inhibition levels against *S. aureus* and *Pseudomonas aeruginosa*, and the diameter of the inhibition zone can reach more than 12.00 mm. Among them, *L. paracasei* M16 had the best inhibitory effect on *S. aureus* and *Bacillus subtilis*, reaching 19.51 ± 0.49 mm and 17.44 ± 0.58 mm, respectively. *L. rhamnosus* M3 (2) had the best inhibitory effect on *Listeria*, reaching 18.05 ± 0.23 mm; *L. paracasei* M5 had the best inhibitory effect on *E. coli* and could reach 14.52 ± 0.26 mm. *P.pentosaceus* M19 had a good inhibitory effect on *Pseudomonas aeruginosa*, and the inhibition zone size reached 21.07 ± 0.19 mm. The results showed a significant difference in the inhibitory effect of varied strains on different indicator bacteria, and the antibacterial effect of different strains varied, pointing to the physiological characteristics of the strain and whether it produced bacteriocin.

### 3.4. Acid Resistance Test Results and Analysis

The effectiveness of probiotics depends on their microbial quality, resistance to harsh gastric environments, and functional characteristics. The human digestive tract is complex, and probiotics need to have a certain ability to survive in its environment [23]. Probiotics must survive for 1.5–2 h at pH 3.0. The viability of microorganisms in simulated gastrointestinal environments is one of the effective indicators for evaluating probiotics. In this experiment, 16 strains of LAB with good antibacterial activity were treated with MRS broth medium with pH 2 and pH 3, and incubated in the anaerobic environment at 37 °C to simulate the acidic conditions of human gastric juice. The results showed that the 16 strains of LAB had good tolerance to acidic conditions at pH 3, but there was a significant difference in tolerance at pH 2. Among the sixteen strains, M5, M3 (1), M2, M7, and M21 (2) showed good acid resistance, so these five LAB were selected for subsequent experiments. Acid resistance results are shown in Table 5.

### 3.5. Bile Salt Tolerance Test Results and Analysis

Bile acids play an important role in digesting food and regulating the balance of intestinal microbial communities. The bile salt concentration in the small intestine of healthy people is between 0.3 and 3.0 g/L. High concentrations of bile salts can antagonize the growth of probiotics, change the permeability of the cell membrane, decompose membrane proteins, and cause cell rupture and death [24]. Five strains of acid-tolerant LAB were tolerated at 0.2% and 0.3% bile salts for 3 h, and the concentrations still reached 10^6^ CFU/mL, they all reach the critical density of live bacteria for functional properties of bacteria, as shown in Table 6. The results showed that when the bile salt content was 0.2%, the survival rates of the five strains were more than 80%, and M3 (1) and M7 had a certain growth trend; when the bile salt content was 0.3%, the survival rates of the five strains were significantly different. Respectively, the survival rates of M3 (1) and M5 were 92.58 ± 10.02 ^a^ and 88.49 ± 0.82 ^c^.

### 3.6. Simulated Gastrointestinal Fluid Tolerance Results and Analysis

The biochemical characteristics of the human gastrointestinal environment are very complex. They contain a variety of different enzymes, which can affect the activity of microbial surface proteins and polysaccharides, and consequently affect the adhesion, colonization, and physiological function of microorganisms in the intestine. The ability of probiotics to survive in the gastrointestinal tract can determine whether LAB can normally play a probiotic role in the human body. In this experiment, five strains of LAB were treated with artificially configured gastric and intestinal juices, and were incubated in an anaerobic environment at 37 °C to simulate the probiotics’ digestive environment in the gastrointestinal tract upon entering the human body to evaluate their viability. As shown in Table 7, the tolerance of M2 and M7 to pepsin in the simulated gastric fluid was poor, failing to reach the viable cell density of 10^6^ CFU/mL, which was consistent with the acid resistance results. The five strains of LAB had good tolerance to trypsin in the simulated intestinal fluid, and the survival rates of M5, M3 (1), and M21 (2) reached more than 95%, 97.89%, 98.63%, and 98.60% respectively.

### 3.7. Adhesion Experiment 

One of the necessary conditions for the long-term physiological function of probiotics in the host intestine is the ability to adhere to and colonize the host intestinal mucosal epithelial cells. They play a role after forming a stable flora in certain parts of the intestine, and adhesion is considered a key step in colonization [25]. At present, the in vitro cell adhesion model is widely used by researchers to evaluate the adhesion of LAB. Caco-2 is a colon cancer cell that can exhibit similar morphology and function to mature intestinal epithelial cells in vitro. It has high flexibility, high repeatability, and low cost, so it is often used as an in vitro model to evaluate the adhesion ability of probiotics [26]. 

**Figure 4:**
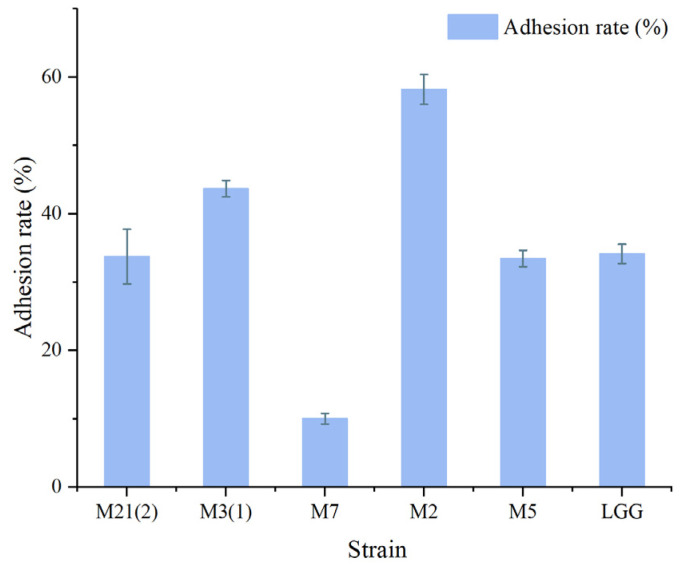
Adhesion rate of LAB.

Five strains of LAB with good tolerance were subjected to adhesion experiments, and the *L. rhamnosus* model strain *L. rhamnosus* GG was used as a positive control. The adhesion results are shown in Figure 4.

It can be seen from the figure that several strains of LAB had certain adhesion abilities with *L. rhamnosus* GG as the positive control (adhesion rate was 34.1 ± 1.4%). Most of the LAB had a similar adhesion rate to *L. rhamnosus* GG, and the adhesion rate of M2 reached 58.2%. The adhesion rates of M21 (2), M3 (1), and M5 were 33.8 ± 4%, 43.7 ± 1.2%, and 33.5 ± 2.4%, respectively. The adhesion rate of M7 was only 10.0%. However, different studies have obtained different results in adhesion ability; therefore, the adhesion experiment can only be used as a reference index, with more in-depth research still needed to clarify the bacterial adhesion ability. 

### 3.8. In Vitro Safety Experiment

From the above experiments, it can be concluded that LAB M5, M3 (1), and M21 (2) had a good comprehensive tolerance to low pH, bile salt, and simulated gastrointestinal fluid, and they exhibited some adhesion ability.

#### 3.8.1. Hemolytic Analysis

Hemolysis is the rupture of red blood cells and the escape of hemoglobin in response to hemolytic toxins and other physicochemical factors. The brownish green agar around the colony indicates α-hemolysis, a transparent circle around the colony points to β-hemolysis, and no change in the agar around the colony means γ-hemolysis (there is no hemolysis). Our results showed that the three strains of LAB were all γ-hemolytic and had no hemolytic effect.

#### 3.8.2. Drug Susceptibility Test Results and Analysis

Antibiotic resistance is an important index parameter for probiotic safety evaluation. The resistance of probiotics can help them better cope with different conditions in the intestine. For example, when the body receives antibiotic treatment, probiotics with good tolerance can colonize effectively, avoid bacterial death, play their role in maintaining the balance of intestinal flora, and promote its recovery and stability [27]. However, with the continuous abuse of antibiotics, more and more LAB are becoming drug resistant or carrying drug resistance genes, which poses a potential safety hazard to human health. The drug-resistant LAB may introduce drug resistance genes to the intestine, allowing other microorganisms in the intestine to obtain drug resistance genes. When these microorganisms are pathogenic, they may affect the therapeutic results; when LAB containing drug resistance genes are potentially pathogenic, antibiotic treatment can also cause additional problems [28].

**Figure 5:**
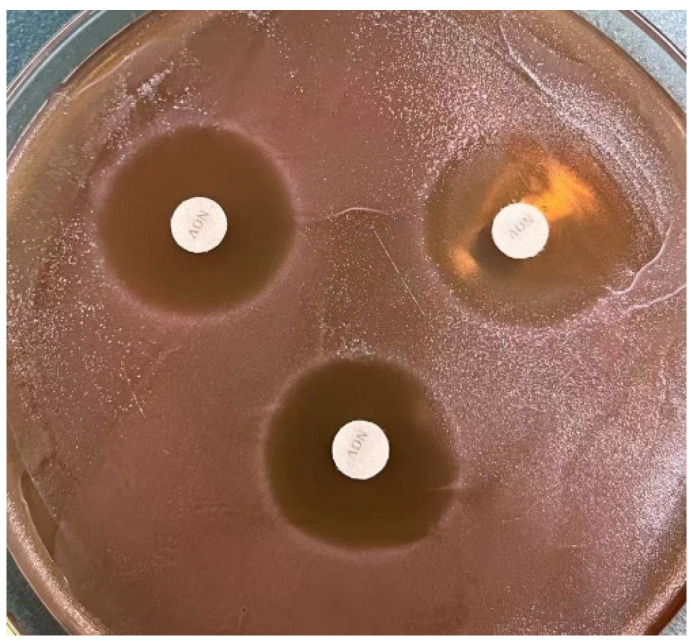
Sensitivity of M5 to novobiocin (NOV).

The results of the drug sensitivity test are shown in Figure 5 and Table 8. The three strains of LAB were sensitive to tetracycline, chloramphenicol, ampicillin, novobiocin, and clindamycin, and resistant to streptomycin, gentamicin, kanamycin, vancomycin, metronidazole, and bacitracin. Most *lactobacilli* are not sensitive to aminoglycoside antibiotics (such as streptomycin and gentamicin) but are sensitive to chloramphenicol. The three strains of LAB are consistent with the drug resistance results of most *lactobacilli* [29]. 

### 3.9. In Vitro Fermentation Test

#### 3.9.1. The Effect of M3 (1) on Intestinal Flora

**Figure 6:**
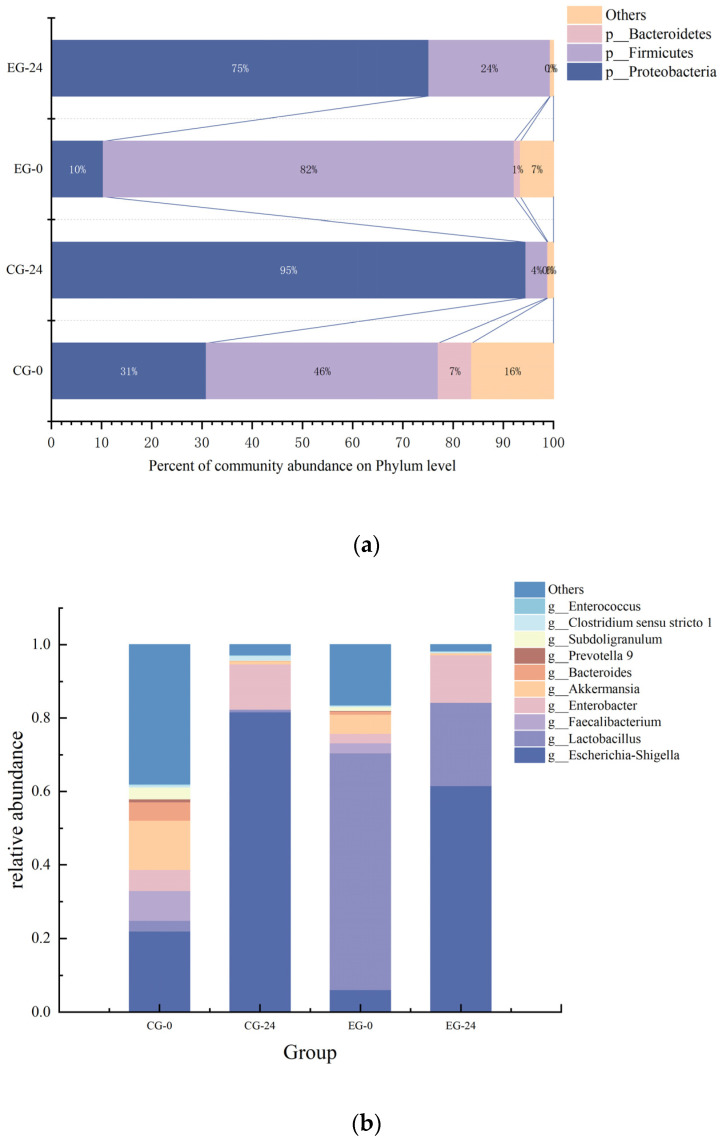
(**a**): Phylum-level species distribution; (**b**): Genus-level species distribution.

Intestinal flora is closely related to host health. The interaction between microorganisms and hosts can stabilize certain internal environments of the human body. A disruption in the balance of microorganisms in the body may result in host metabolic dysfunction and chronic inflammation, leading to UC, irritable bowel syndrome, food allergy, gastritis and peptic ulcer, cardiovascular disease, and gastrointestinal cancer [30]. In order to study the effect of M3 (1) on the intestinal flora of IBD patients, we used high-throughput sequencing analysis and QIME software to obtain the bacterial composition and abundance of each sample at each classification level. The community composition analysis diagram visually illustrates the community structure at the phylum level (Figure 6a) and the genus level (Figure 6b). As shown in the figure, all groups were composed mainly of *Proteobacteria*, *Firmicutes*, and *Bacteroidetes* at the phylum level, while the proportion of *Actinobacteria* is very small, which was similar to cases reported in enteritis patients. At 0 h of in vitro fermentation, *Proteobacteria* and *Firmicutes* were significantly different between the control group (CG) and the experimental group (EG) with M3 (1). The proportion of *Firmicutes* in the EG group was significantly higher than in the CG group, and the relative abundance reached 82%. *Proteobacteria* accounted for 31% and 10% of the CG group and EG group, respectively. With the progress of fermentation, the proportion of *Proteobacteria* increased rapidly and became the dominant flora. At 24 h of in vitro fermentation, the relative abundance of the CG group reached 95%, and that of the EG group was 73%. At this time, the *Firmicutes* of the EG group were significantly higher than in the of CG group (*p* < 0.05), accounting for 21%, which was about five times that of the CG group. The low expression of *Lactobacillus*, *Bifidobacterium*, and *fecal bacilli* in fecal samples, and the abnormally rapid growth of *Proteobacteria*, mean that the host intestinal flora was unbalanced, a result that can be used as a potential diagnostic criterion for enteritis diseases and prove the reliability of this experimental sample. Therefore, adding M3 (1) can, to some extent, inhibit the growth of *Proteobacteria* in the intestinal environment of IBD patients, minimize their increase, and improve the abundance of *Firmicutes*. 

In a further comparison of the intestinal microflora composition in the fermentation broth, shown in the figure, at the genus level, the intestinal microflora was mainly composed of *Escherichia coli -Shigella* (*E. coli/Shigella*), *Lactobacillus*, *Enterobacter*, *Akkermansia*, and *Bacteroides*. *E. coli/Shigella*, *Akmannia*, *Subdoligranulum*, *Enterobacter*, and *Bacteroides* levels in the CG group were higher than in the EG group, while *Lactobacillus* was higher in the EG group, which was related to the addition of M3 (1). The differences at the genus level are consistent with the trend of the analysis results at the gate level, further verifying the experiment’s depth and credibility. It can be seen from Figure 6b that with the progress of fermentation, *E. coli/Shigella* dominated the sample and reached 82% at 24 h in the CG group. Some *E. coli/Shigella* affect the intestinal mucosal barrier function of the host, which is a critical microorganism leading to diarrhea [31]. However, in the EG group, the relative abundance of *E. coli/Shigella* was only 62%, 20% lower than in the CG group, which effectively inhibited the *E. coli/Shigella*. *Lactobacillus* can inhibit the growth of *Salmonella typhimurium*, enterohemorrhagic *E. coli*, and *Shigella* by secreting acidic substances such as lactic acid or nonacidic bacteriocins [32]. In the host, it can also inhibit the growth of intestinal pathogenic microorganisms by competing for nutrients and colonization sites.

In summary, M3 (1) has a significant inhibitory effect on the harmful microorganism *E. coli/Shigella* in the intestine without affecting the indigenous microorganisms such as *Enterobacter*, and to some extent, it promotes the growth of *Lactobacillus*. It has a positive regulatory effect on the intestinal flora of IBD patients.

#### 3.9.2. Correlation Analysis between pH and SCFAs

It can be seen from the figure that as the fermentation progressed, the formation of acidic substances led to a change in the fermentation medium pH, and the pH of the in vitro fermentation broth changed significantly. The initial pH was about 7.2. After 24 h of fermentation, the pH decreased significantly. The EG group was significantly lower than the CG group because of the growth and metabolism of the intestinal flora. The fermentation of probiotics such as LAB produced a large amount of short-chain fatty acids, which reduced the pH value and indirectly reflected the changes in intestinal microflora. 

**Figure 7:**
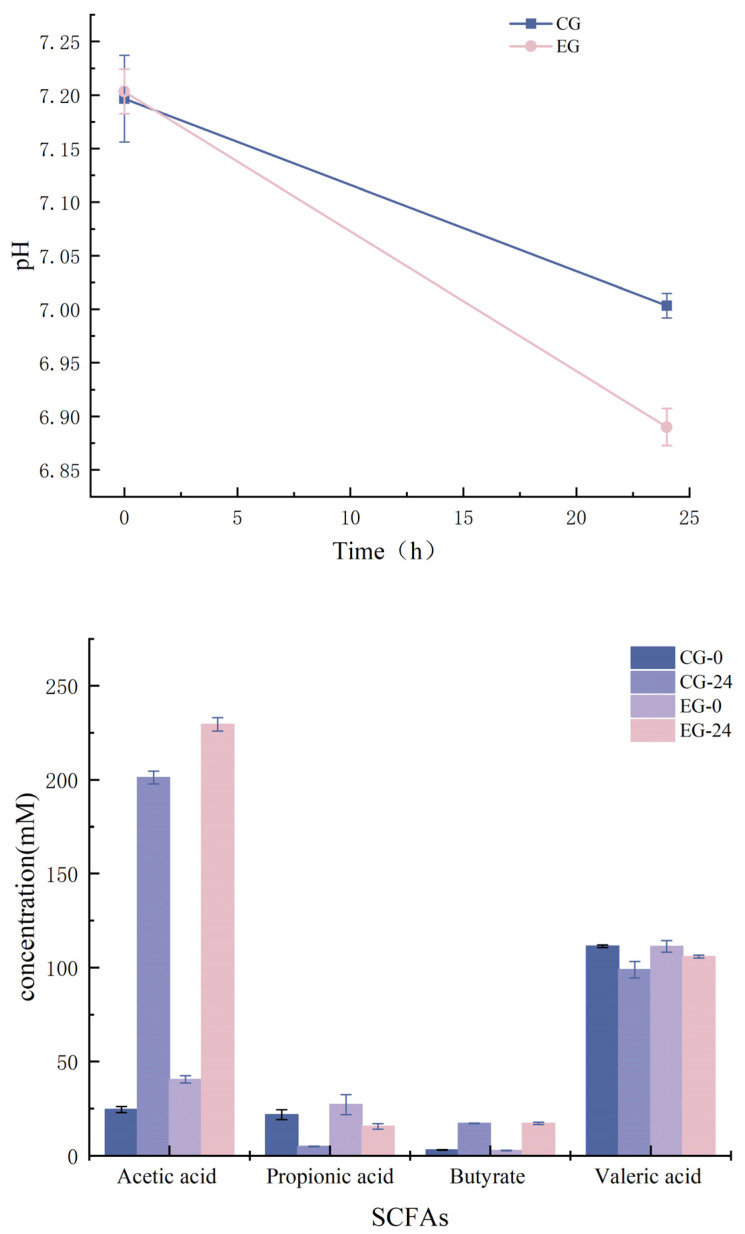
pH and SCFA concentration during in vitro fermentation (*p* < 0.05).

SCFAs are formed by intestinal microbes fermenting dietary fiber, carbohydrates, and other plant nutrients in the intestine, and are an essential energy source for peripheral tissues such as intestinal microbes and colonic epithelium [33]. The catabolism of carbohydrates by human intestinal microorganisms mainly produces three SCFAs: acetic acid, propionic acid, and butyric acid [34]. To understand the effect of M3 (1) on the production of SCFAs by fecal microorganisms in IBD patients, this study analyzed the fecal mixture of four groups at each fermentation time point using GC-MS, with acetic acid (C4) as a standard for detecting acetic acid, propionic acid, butyric acid, caproic acid, and valeric acid. The results showed that the SCFAs detected in different groups of feces were mainly acetic acid, propionic acid, butyric acid, and valeric acid. The proportion of acetic acid was the highest, followed by valeric acid, then propionic acid, and the proportion of butyric acid was the lowest. The type of SCFAs and the concentration produced during fermentation are shown in Figure 7.

After 24 h of fermentation, the concentrations of acetic acid and butyric acid in the two groups increased significantly, while the concentrations of propionic acid and valeric acid decreased slightly. Compared with the CG group, the concentration of acetic acid and valeric acid in the EG group increased significantly (*p* < 0.05), and the concentration of propionic acid was significantly higher than that in the CG group at 24 h of fermentation. The measured concentration of butyric acid was similar in the two treatments, and the EG group was slightly higher than the CG group. The concentration of SCFAs was positively correlated with the change in pH value. It has been reported that SCFAs are beneficial to human health. The acetic acid content in the four SCFAs were the highest, followed by valeric acid. Acetic acid is usually produced by a variety of microorganisms and reaches its highest concentration in the intestinal lumen [35]. Propionic acid is the primary metabolite of *Bacteroides*, which can affect the metabolism of the host liver and cholesterol [36], reduce serum and cholesterol levels [37], and prevent diet-induced obesity. Butyric acid is mainly produced by the metabolism of *Bacteroidetes* and *Firmicutes*, which is closely related to intestinal health [38]. It is the energy source of colonic epithelium and indirectly affects the metabolism of sugars and lipids by participating in gluconeogenesis, ketone body formation, and triglyceride synthesis [39]. There was no significant difference in the concentration of butyric acid between the EG and CG groups, which was consistent with the analysis of the proportion of *Enterobacter* at the genus level. Valeric acid is produced by the metabolism of microorganisms such as *Lactobacillus* and *Bacteroides*, and can be produced by the fermentation of dietary fiber by intestinal microorganisms. Valeric acid can affect immune regulation, reduce the body’s inflammatory response, improve blood pressure, and reduce the occurrence of eczema [40]. At 24 h of fermentation, the valeric acid content in the EG group increased significantly, which was consistent with the increase in *Lactobacillus* proportion. 

These results suggest that the production of SCFAs such as acetic acid may change the pH of the intestinal environment and affect the intestinal microflora. The intervention of M3 (1) can change the intestinal flora, promote the production of SCFAs such as acetic acid and propionic acid, reduce the pH environment, reduce the colonization of harmful microorganisms, improve intestinal health, and reduce the occurrence of intestinal inflammation. 

### 3.10. Flavor Analysis 

The M3 (1) strain with better comprehensive ability was used for the milk fermentation test, and its pH and volatile flavor substances were detected and analyzed.

#### 3.10.1. pH Changes 

**Figure 8:**
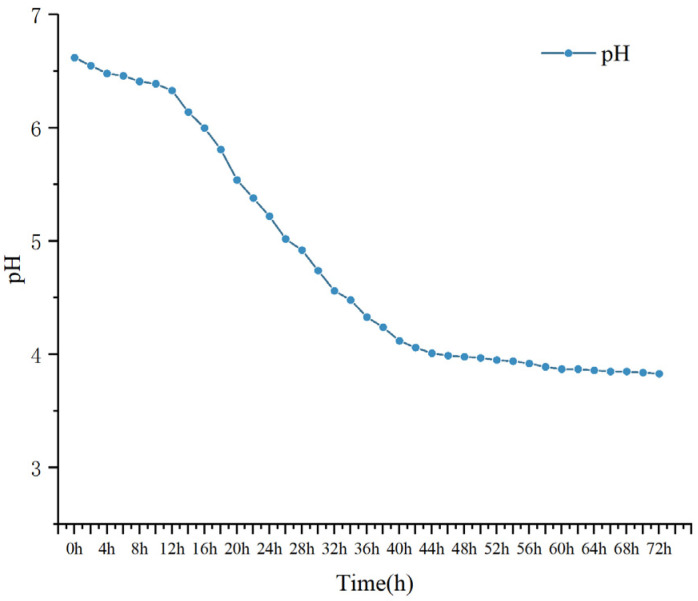
The trend of pH of M3 (1)-fermented milk.

The pH value of milk was recorded every two hours form the access to M3 (1). The results are shown in Figure 8. It can be seen from the figure that with the extension of the access time of LAB, the pH of fermented milk decreased significantly, and it entered the end of fermentation at about the 42 h point. As the pH of the fermented milk decreased, microorganisms consumed sugars and fatty acids in the early stages of fermentation to produce low-molecular-weight acids [41], which reduced the pH to the end of fermentation. 

#### 3.10.2. Analysis of Volatile Components 

**Figure 9:**
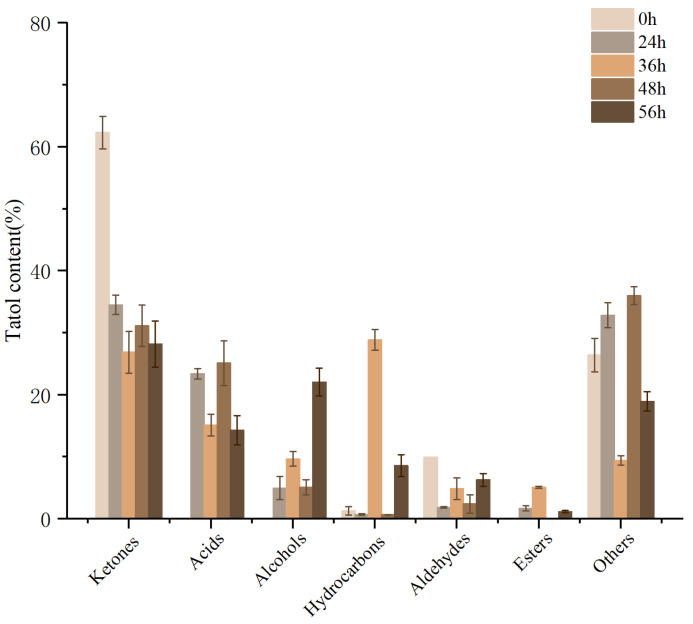
Changes in components of milk during fermentation.

The unique flavor of yogurt is composed of a complex mixture of lactic acid and aromatic compounds. Free amino acids produced by protein decomposition after inoculating LAB can be converted into flavor compounds, including ammonia, amines, aldehydes, phenols, indoles, and alcohols [42]. M3 (1)-fermented milk conforms to the fermentation properties of *L. rhamnosus* and brings a good flavor to the milk. There are significant differences in volatile metabolites at different time points. A total of 41 volatile flavor substances were detected by GC-MS (Table 9), including esters (3), ketones (8), aldehydes (4), acids (3), alcohols (3), hydrocarbons (11), and other compounds (11). Among them, the most volatile flavor substances in the fermented milk samples were hydrocarbons, ketones, acids, and alcohols as the main flavor contributors (Figure 9).

Ketones are the main volatile organic compounds in fermented milk at all stages. Eight ketones were identified, among which 2-nonanone and 2-heptanone were the most abundant, which significantly affected the flavor of M3 (1)-fermented milk and provided its cream flavor [43]. Acetoin was found at 36 h and 48 h of fermentation. *L. rhamnosus* can metabolize lactose and citrate to produce diacetyl, and further oxidize to produce acetoin [44]. The production of acetoin can add pleasant smells such as butter and caramel to fermented milk [45]. No acids were detected in unfermented milk, meeting the quality standards. As the fermentation progressed, the acids in milk increased significantly. These acids are usually derived from lipolysis, proteolysis, or lactose fermentation [46]. Acetic acid is a common flavor substance in fermented milk, which has a significant influence on the taste of fermented milk. *L. rhamnosus* belongs to heterogeneous fermentation; in the heterogeneous fermentation pathway, glucose and some galactose molecules in milk can be converted into glyceryl 3-phosphate and acetyl CoA by phosphorylase. Some acetyl CoA can be reduced to ethanol by NADH [47]. Under oxidation conditions, a large amount of acetyl can be converted into acetic acid [48]. A high concentration of acetic acid has a spicy and irritating odor, which has a negative effect on fermented milk. This may be related to the termination of LAB fermentation and the occurrence of esterification [49], making the flavor softer. In addition to the common acetic acid, caproic and octanoic acids were also detected at 36 h of fermentation. These two acids can give the fermented milk a cheese, caramel, and floral flavor, increasing the characteristic flavor of M3 (1)-fermented milk. In addition to the main volatile organic compounds that affect the flavor of regular yogurt [50], we found that nonanal and heptanal were produced in milk fermented with M3 (1), which may be formed by the metabolic decarboxylation of amino acids such as methionine and phenylalanine by M3 (1), and can also be formed by the secondary oxidation of fatty acids [51]. They increase fermented milk’s sweet, floral, citrus, and grass aroma [52]. Esters are odor-active compounds that can be formed in the reaction of alcohols with organic acids and may also be produced by coupling with CoA [53]. M3 (1)-fermented milk mainly produces isocyanates, octyl formate, and methyl formate. Although its mass fraction is not high, it significantly contributes to the taste of fermented milk and produces its fruit flavor [54].

## 4. Discussion

One hundred and three strains of LAB were isolated from different samples. They were identified as 14 species: *L. plantarum*, *L. brevis*, *Staphylococcus* sp., *L. paracasei*, *P. pentosaceus*, *E. avium*, *B. breve*, *B. crudilactis*, *S. salivarius*, *E. faecium*, *W. cibaria*, *L. rhamnosus*, *B. psychraerophilum*, and *L. parabuchneri*. The results showed that the species of LAB isolated from different sources differed because of the samples’ nutritional composition and pH environment.

The antibacterial ability, acid and bile salt resistance, and simulated gastrointestinal fluid resistance of the same strain isolated from different sources were also different. The screened LAB had a good inhibitory effect on the growth of *S. aureus* and *Pseudomonas aeruginosa*, which could reach 12.00 mm. The inhibitory effect on other strains differed. The five strains M5, M3 (1), M2, M7, and M21 (2) had good acid resistance. The survival rate of M3 (1) and M5 incubated in 0.3 % bile salt for 3 h was above 85 %. M5 and M3 (1) showed good tolerance to simulated gastrointestinal fluid, and their survival rate was higher than 95%.

The adhesion rate of M3 (1) was higher than that of *L. rhamnosus* GG, and their comprehensive evaluation was better. Therefore, basic research on in vitro fermentation and the application of M3 (1) was carried out, and it was found that M3 (1) could change the intestinal flora of IBD patients. Without affecting other probiotics, it could effectively inhibit the growth of *E. coli/Shigella*, promote the production of SCFAs such as acetic acid and propionic acid, and reduce the pH of the internal environment. Milk fermented with M3 (1) could produce a classic and pleasant flavor. Therefore, *L. rhamnosus* M3 (1) is expected to be used in microecological preparation and probiotic auxiliary starter cultures, which can survive and colonize in the digestive system, effectively inhibit the growth of harmful microorganisms in the intestine, regulate the intestinal flora, promote intestinal health, and enhance the probiotic value of fermented milk.

Finding LAB with probiotic functions from different environments can enrich the LAB resource library. The genetic, physiological, metabolic, and fermentation characteristics of LAB from different sources were systematically studied, and the functional characteristics of different strains were clarified. For example, *L. plantarum* Z1 and Z2 strains obtained from fermented vegetables had poor antibacterial ability compared with single tolerance ability but had an outstanding inhibitory ability against *Bacillus subtilis*. In the fermentation process, it can effectively inhibit the growth of *Bacillus* and improve the flavor and fermentation performance of fermented vegetables, which provides a new opportunity for preserving and safely controlling heat-sensitive foods.

Probiotic LAB are an important part of the larger healthcare context. Their biodiversity can be used as an important scientific basis to conduct targeted research and development of probiotics, synbiotics, metazoans, and other related health products, and to obtain abundant LAB resources. At the same time, LAB derived from traditionally fermented foods and healthy humans have relatively safe biological characteristics. They have broad application potential as biological expression vectors for functional components and chassis cells for synthetic biology. Therefore, constructing and developing LAB germplasm resources is a common mission of scientific and technological workers with common interests in this field.

## Figures and Tables

**Table 1:** Main reagents and materials.

Name of Material or Reagent	Source or Manufacturer
*Staphylococcus aureus* (*S. aureus*) ATCC 6538	Center for Microbial Species Preservation, Chinese Academy of Sciences (Beijing, China)
*Listeria monocytogenes* (*L. monocytogenes*) ATCC 19115	
*Escherichia coli* (*E. coli*) CGMCC 9181	
*Pseudomonas aeruginosa*	Laboratory of Food Science and Technology College of Hunan Agricultural University (Changsha, China)
*Bacillus subtilis*	
Soybean peptone, tryptone	Thermo Fisher Oxoid (Shanghai, China)
MRS agar medium, MRS broth, technical agar powder	Guangdong Huankai Microbial Technology Co., Ltd. (Guangzhou, China)
Blood agar plate, Gram staining kit	
Pig bile salt	
Pepsin	Beijing Soleibao Technology Co., Ltd. (Beijing, China)
Sensitivity paper	Hangzhou Microbial Reagent Co., Ltd. (Hangzhou, China)
DNA Bacterial Extraction Kit	Hangzhou Beiwo Medical Technology Co., Ltd. (Hangzhou, China)
Trypsin	Sinopharm Chemical Reagent Co., Ltd. (Shanghai, China)
Fetal calf serum	Biological Industries Co., Ltd. (Guangzhou, China)
Dulbecco’s modified eagle medium high glucose medium	
Trypsin-EDTA	
Short-chain fatty acid standard	Shanghai Macklin Biochemical Co., Ltd. (Shanghai, China)

**Table 2:** Drug resistance judgment standard reference.

Item	Drug Category	Drug Content on Paper (μg/Piece)	Evaluation Criteria (mm)		
			S	I	R
Clindamycin	Lincomycin class	2.0	≥21.00	15.00–20.00	≤14.00
Streptomycin	Aminoglycosides	10.0	≥15.00	12.00–14.00	≤11.00
Gentamicin	Aminoglycosides	10.0	≥15.00	13.00–14.00	<12.00
Kanamycin	Aminoglycosides	30.0	≥18.00	14.00–17.00	≤13.00
Vancomycin	Polypeptide Class	30.0	>17.00	14.00–17.00	<14.00
Tetracycline	Tetracycline class	30.0	≥19.00	15.00–18.00	≤14.00
Chloramphenicol	β-Lactams	30.0	≥18.00	13.00–17.00	≤12.00
Ampicillin	Penicillin class	10.0	≥17.00	14.00–16.00	≤13.00
Metronidazole	Nitroimidazole class	5.0	≥16.00	11.00–15.00	≤10.00
Bacitracin	Polypeptide Class	0.4	≥10.00	-	<10.00
Cotrimoxazole	Sulfonamides	25.0	≥16.00	10.00–16.00	<10.00
Neomycin	Coumarin Class	30.0	≥17.00	13.00–16.00	<12.00
Ofloxacin	Quinolones	5.0	≥16.00	13.00–15.00	≤12.00

S (sensitive): recommended doses achieve good inhibition; I (intermediary): strains of antimicrobial drugs are close to the levels achievable in blood and tissues, but the therapeutic efficiency response rate may be lower than that of sensitive strains; R (resistant): normal doses have no significant inhibitory effect.

**Table 3:** Partial comparison results of 16SrRNA sequence homology of screening isolates from different sources.

Strain	Source	Strain Name	Strain Number	Login Number	Similarity%
E5	Fermented bamboo shoots	*L. plantarum*	*L. plantarum strain* NRRL B-14768	NR_042394.1	99.66%
F9	Fermented bamboo shoots	*L. plantarum*	*L. plantarum strain* 3335	MT613628.1	99.86%
C2	Fermented bamboo shoots	*L. plantarum*	*L. plantarum strain* BCH-2	KX388383.1	99.86%
A1	Fermented bamboo shoots	*L. plantarum*	*L. plantarum strain* JCM 1149	NR_115605.1	99.79%
G3	Fermented bamboo shoots	*L. brevis*	*L. brevis strain* 23	MH681601.1	99.86%
C4	Fermented bamboo shoots	*L. brevis*	*L. Brevis strain* ATCC 14869	NR_044704.2	99.52%
D10	Fermented vegetables	*L. brevis*	*L. brevis strain* ATCC 14869	NR_044704.2	99.45%
F10	Fermented vegetables	*L. brevis*	*L. brevis strain* NWAFU1541	MG551215.1	99.86%
D6	Fermented cowpeas	*L. plantarum*	*L. plantarum strain* 3784	MT538636.1	99.86%
D11	Fermented cowpeas	*L. plantarum*	*L. plantarum strain* thankcomeLP1	MZ045749.1	99.86%
G5	Fermented bamboo shoots	*L. plantarum*	*L. plantarum strain* TMPC 33321	OM265411.1	100.00%
G7	Fermented bamboo shoots	*L. plantarum*	*L. plantarum strain* LAB-12	MW928452.1	99.93%
F11(2)	Fermented vegetables	*L. brevis*	*L. brevis strain* 1997	MT597799.1	99.80%
B11	Fermented vegetables	*L. plantarum*	*L. plantarum strain* Sourdough_B11	MG754629.1	99.86%
C7	Pickled Chili	*L. plantarum*	*L. plantarum strain* MLG4-3-1	MT473372.1	100.00%
C1	Fermented bamboo shoots	*L. brevis*	*L. brevis strain* 1997	MT597799.1	99.86%
Fa16	Infant feces	*L. paracasei*	*L. paracasei strain* HBUAS52231	MH472956.1	92.55%
Fb5	Feces of obese people	*Enterococcus avium* (*E. avium*)	*E. avium strain* MG4610	ON631294.1	96.73%
Fa17	Infant feces	*Bifidobacterium breve* (*B. breve*)	*B. breve strain* 1176	MT573641.1	92.27%
Fa1	Infant feces	*L. paracasei*	*L. paracasei strain* 5601	MT510433.1	97.93%
F2	Feces of obese people	*L. paracasei*	*L. paracasei strain* 6582	MT463826.1	98.24%
F5	Feces of obese people	*B. crudilactis*	*B. crudilactis strain* C4/12B	MG639889.1	96.30%
F3	Feces of obese people	*S. salivarius*	*S. salivarius strain* 1740	MT597596.1	96.28%
F9	Feces of obese people	*E. faecium*	*E. faecium strain* CDCP37	MT814619.1	95.54%
F6	Feces of obese people	*Weissella cibaria* (*W. cibaria*)	*W. cibaria strain* M.D.W.YAN2-10	JF690894.1	97.42%
F8	Feces of obese people	*E. faecium*	*E. faecium isolate* NSY	EU047802.1	96.17%
M7	Raw milk	*L. rhamnosus*	*L. rhamnosus strain* 2949	MT611888.1	96.08%
M3(1)	Raw milk	*L. rhamnosus*	*L. rhamnosus strain* YIT 0105 (=ATCC 7469)	AB008211.1	97.06%
M2	Raw milk	*P.pentosaceus*	*P.pentosaceus s strain* F8S2	KF245570.1	97.62%
M19	Raw milk	*P.pentosaceus*	*P.pentosaceus strain* 6359	MT463768.1	97.04%
M8(1)	Raw milk	*L. parabuchneri*	*L. parabuchneri strain* B3BL14G	MW700852.1	97.64%
M14	Raw milk	*B. crudilactis*	*B. crudilactis strain* C4/12B strain C4/12B	MG639889.1	97.17%
M9	Raw milk	*Bifidobacterium psychraerophilum* (*B. psychraerophilum*)	*B. psychraerophilum strain* DA-50B	KJ128206.1	96.26%
M6	Raw milk	*L. paracasei*	*L. paracasei strain* 6493	MT515921.1	97.91%
M5	Raw milk	*L. paracasei*	*L. paracasei strain* 4888	MT505631.1	97.53%
M3(2)	Raw milk	*L. paracasei*	*L. paracasei strain* 6522	MT515950.1	97.70%
M2(2)	Raw milk	*B. crudilactis*	*B. crudilactis strain* C4/12B	MG639889.1	96.70%
M8(2)	Raw milk	*L. parabuchneri*	*L. parabuchneri strain* 2012	MT604605.1	98.49%

**Table 4:** Inhibition results of the screened LAB (top 16 strains).

Strain	*S. aureus* (mm)	*L. monocytogenes* (mm)	*E. coli* (mm)	*Bacillus subtilis* (mm)	*Pseudomonas aeruginosa* (mm)
M16	19.51 ± 0.49	10.97 ± 0.49	-	17.44 ± 0.58	17.69 ± 1.60
M6	19.45 ± 0.51	-	10.32 ± 0.00	10.44 ± 0.14	18.17 ± 0.47
FU	17.23 ± 1.55	10.16 ± 0.00	10.37 ± 0.21	12,70 ± 0.00	18.80 ± 0.06
F2	17.57 ± 0.27	11.36 ± 0.00	-	9.70 ± 0.16	14.79 ± 0.89
M11 (2)	16.99 ± 0.33	11.02 ± 0.34	9.23 ± 0.41	12.55 ± 3.77	17.46 ± 0.24
M7	17.02 ± 0.00	13.79 ± 0.13	8.02 ± 0.00	-	15.37 ± 0.27
M5	16.13 ± 0.07	11.37 ± 0.05	14.52 ± 0.26	12.16 ± 1.89	18.59 ± 0.49
M3 (2)	16.02 ± 0.00	18.05 ± 0.23	8.93 ± 0.11	-	18.68 ± 0.48
M19	15.56 ± 0.46	14.25 ± 0.33	-	-	21.07 ± 0.19
M12	14.83 ± 0.37	15.42 ± 0.10	-	16.21 ± 1.31	16.54 ± 0.06
M21 (2)	15.10 ± 0.04	-	-	15.12 ± 1.14	20.45 ± 2.61
M3 (1)	15.04 ± 0.06	14.48 ± 0.18	9.60 ± 0.20	13.41 ± 1.39	20.09 ± 0.21
M12 (1)	14.87 ± 0.37	15.75 ± 0.27	-	11.77 ± 0.21	14.99 ± 0.49
Z2	13.11 ± 0.61	11.63 ± 0.27	-	15.53 ± 0.55	14.33 ± 1.35
D2	13.57 ± 0.23	11.75 ± 0.29	-	-	12.05 ± 0.71
Z1	12.84 ± 0.18	9.79 ± 0.29	-	16.05 ± 0.89	18.80 ± 0.10

Oxford cup size: inner diameter 6 mm, outer diameter 7 mm, table: - indicates that the inhibition circle is less than 1 mm, negligible, inhibition is negative.

**Table 5:** 16 strains of LAB acid resistance results.

Strain	pH = 2		PH = 3	
	0 h (l g CFU/mL)	3 h (l g CFU/mL)	0 h (l g CFU/mL)	3 h (l g CFU/mL)
M3 (1)	6.53 ± 0.090 ^a^	5.56 ± 0.100 ^c^	6.26 ± 0.055 ^a^	6.28 ± 0.078 ^b^
M5	7.23 ± 0.416 ^cd^	7.11 ± 0.090 ^f^	7.28 ± 0.072 ^c^	7.27 ± 0.046 ^h^
M3 (2)	7.25 ± 0.092 ^cd^	ND ^a^	7.12 ± 0.115 ^c^	6.80 ± 0.118 ^f^
M7	6.75 ± 0.075 ^b^	4.20 ± 0.020 ^b^	7.15 ± 0.070 ^c^	6.37 ± 0.076 ^c^
M16	7.18 ± 0.072 ^cd^	ND ^a^	7.24 ± 0.049 ^c^	6.63 ± 0.058 ^e^
M6	7.79 ± 0.011 ^e^	ND ^a^	7.19 ± 0.061 ^c^	6.87 ± 0.012 ^f^
M11 (2)	6.43 ± 0.117 ^a^	ND ^a^	6.64 ± 0.036 ^b^	6.23 ± 0.006 ^bc^
M2	7.31 ± 0.021 ^d^	6.28 ± 0.062 ^d^	7.35 ± 0.105 ^c^	7.02 ± 0.015 ^g^
M19	6.81 ± 0.067 ^b^	ND ^a^	6.64 ± 0.288 ^b^	6.19 ± 0.055 ^b^
M12 (1)	6.41 ± 0.031 ^a^	ND ^a^	6.35 ± 0.104 ^a^	6.23 ± 0.055 ^bc^
M21 (2)	7.12 ± 0.093 ^c^	6.68 ± 0.387 ^e^	7.23 ± 0.110 ^c^	6.21 ± 0.025 ^g^
Z1	7.13 ± 0.105 ^c^	ND ^a^	7.29 ± 0.077 ^c^	6.20 ± 0.025 ^b^
Z2	7.26 ± 0.164 ^cd^	ND ^a^	7.34 ± 0.115 ^c^	6.21 ± 0.058 ^bc^
D2	7.13 ± 0.095 ^a^	ND ^a^	6.37 ± 0.066 ^a^	6.21 ± 0.081 ^b^
F2	6.84 ± 0.095 ^c^	ND ^a^	6.77 ± 0.110 ^b^	5.63 ± 0.031 ^a^
FU	6.98 ± 0.053 ^b^	ND ^a^	6.61 ± 0.061 ^b^	6.41 ± 0.031 ^d^

ND: Not detected. In the same column value, different shoulder letters indicate significant difference (*p* < 0.05).

**Table 6:** Bile salt tolerance of LAB.

Strain	0.20%		0.30%	
	0 h (l g CFU/mL)	3 h (l g CFU/mL)	0 h (l g CFU/mL)	3 h (l g CFU/mL)
M3 (1)	6.32 ± 0.023 ^a^	6.04 ± 0.012 ^a^	6.33 ± 0.036 ^a^	6.32 ± 0.080 ^a^
M2	7.30 ± 0.067 ^d^	7.28 ± 0.056 ^d^	6.61 ± 0.034 ^b^	6.34 ± 0.024 ^b^
M7	6.49 ± 0.015 ^b^	6.49 ± 0.004 ^b^	7.49 ± 0.010 ^c^	6.84 ± 0.034 ^b^
M5	7.10 ± 0.007 ^c^	7.08 ± 0.003 ^c^	7.11 ± 0.031 ^d^	7.06 ± 0.027 ^c^
M21 (2)	7.17 ± 0.052 ^c^	7.11 ± 0.000 ^c^	7.06 ± 0.554 ^e^	7.48 ± 0.005 ^d^

In the same column value, different shoulder letters indicate significant difference (*p* < 0.05).

**Table 7:** Tolerance of LAB simulated gastrointestinal fluid.

Strain	Simulated Gastric Juice (pH = 2)		Simulated Intestinal Fluid (pH = 6.8)	
	0 h (l g CFU/mL)	3 h (l g CFU/mL)	0 h (l g CFU/mL)	3 h (l g CFU/mL)
M5	8.65 ± 0.006 ^a^	6.46 ± 0.012 ^c^	5.23 ± 0.035 ^c^	5.12 ± 0.017 ^c^
M3 (1)	8.79 ± 0.014 ^d^	6.33 ± 0.010 ^b^	5.11 ± 0.024 ^d^	5.04 ± 0.113 ^d^
M21 (2)	9.80 ± 0.018 ^c^	7.23 ± 0.018 ^d^	5.01 ± 0.017 ^b^	4.94 ± 0.017 ^c^
M2	8.07 ± 0.032 ^a^	4.27 ± 0.002 ^a^	3.51 ± 0.001 ^a^	2.87 ± 0.130 ^b^
M7	8.09 ± 0.042 ^a^	ND ^e^	3.48 ± 0.011 ^a^	2.65 ± 0.171 ^a^

ND: Not detected. The same column numerical shoulder letters showed significant differences (*p* < 0.05).

**Table 8:** Drug sensitivity test results.

Item	Size of Inhibition Zone of M3 (1) (mm)	Sensitive Level	Size of Inhibition Zone of M21 (2) (mm)	Sensitive Level	Size of Inhibition Zone of M5 (mm)	Sensitive Level
Clindamycin	31.53 ± 0.34	S	29.06 ± 0.58	S	18.35 ± 0.97	I
Streptomycin	9.47 ± 0.52	R	7.00 ± 0.00	R	7.94 ± 0.58	R
Gentamicin	11.29 ± 0.43	R	9.967 ± 0.613	R	10.29 ± 0.99	R
Kanamycin	9.95 ± 0.62	R	10.69 ± 0.27	R	7.00 ± 0.00	R
Vancomycin	7.00 ± 0.00	R	7.00 ± 0.00	R	7.00 ± 0.00	R
Tetracycline	34.73 ± 0.87	S	21.90 ± 0.62	S	17.91 ± 0.33	S
Chloramphenicol	33.74 ± 0.23	S	31.43 ± 0.02	S	23.76 ± 1.56	S
Ampicillin	22.70 ± 0.50	S	16.92 ± 0.16	I	27.88 ± 0.86	S
Metronidazole	7.00 ± 0.00	R	7.00 ± 0.00	R	8.90 ± 0.78	R
Bacitracin	7.00 ± 0.00	R	7.00 ± 0.00	R	7.00 ± 0.00	R
Cotrimoxazole	7.00 ± 0.00	R	7.00 ± 0.00	R	13.31 ± 0.43	I
Neomycin	32.34 ± 0.16	S	16.89 ± 0.14	I	23.47 ± 2.37	S
Ofloxacin	17.50 ± 0.02	S	8.16 ± 0.27	R	7.00 ± 0.00	R

As shown in the notes to Table 2 above.

**Table 9:** Five fermentation stages of fermented milk were detected by GC-MS.

NO	Name	Formula	CAS	Relative Amount (%)				
				0 h	24 h	36 h	48 h	56 h
1	2-Nonanone	C_9_H_18_O	821-55-6	8.928 ± 0.833 ^ab^	10.495 ± 0.265 ^b^	8.897 ± 0.881 ^ab^	8.3663 ± 0.969 ^a^	10.528 ± 1.221 ^b^
2	2-Butanone, 3-methyl-	C_5_H_10_O	563-80-4	1.674 ± 1.176 ^b^	ND	ND	0.472 ± 0.041 ^a^	ND
3	Pyrolo[3,2-d]pyrimidin-2,4(1H,3H)-dione	C_6_H_5_N_3_O_2_	65996-50-1	ND	ND	ND	0.477 ± 0.049 ^b^	ND
4	2-Undecanone	C_11_H_22_O	112-12-9	ND	ND	2.087 ± 0.363 ^c^	ND	1.433 ± 0.492 ^b^
5	Isophorone	C_9_H_14_O	78-59-1	ND	ND	3.003 ± 0.444 ^b^	ND	4.67 ± 0.459 ^c^
6	1-Pentanone, 1-(4-methylphenyl)-	C_12_H_16_O	1671-77-8	ND	ND	0.433 ± 0.335 ^b^	ND	ND
7	Acetoin	C_4_H_8_O_2_	513-86-0	ND	ND	ND	0.480 ± 0.052 ^b^	0.367 ± 0.169 ^b^
8	2-Heptanone	C_7_H_14_O	110-43-0	51.697 ± 0.624 ^d^	24.010 ± 1.297 ^c^	12.437 ± 1.325 ^a^	21.320 ± 2.236 ^b^	11.193 ± 1.376 ^a^
9	Acetic acid	C_2_H_4_O_2_	64-19-7	ND	23.368 ± 0.830 ^c^	9.790 ± 0.840 ^b^	25.107 ± 3.583 ^c^	14.278 ± 12.378 ^bc^
10	Hexanoic acid	C_6_H_12_O_2_	142-62-1	ND	ND	2.868 ± 0.636 ^b^	ND	ND
11	Octanoic acid	C_8_H_16_O_2_	124-07-2	ND	ND	2.457 ± 0.289 ^b^	ND	ND
12	2-Heptanol	C_7_H_16_O	543-49-7	ND	4.980 ± 4.888 ^b^	9.680 ± 9.183 ^c^	5.100 ± 6.227 ^b^	20.243 ± 16.089 ^d^
13	2-Nonanol	C_9_H_20_O	628-99-9	ND	ND	ND	ND	1.827 ± 0.129^b^
14	Longifolene	C_15_H_24_	475-20-7	1.310 ± 0.156 ^c^	0.723 ± 0.136 ^b^	ND	0.677 ± 0.038 ^b^	0.813 ± 0.095 ^b^
15	Benzene, 1-methyl-3-(1-methylethyl)-	C_10_H_14_	535-77-3	ND	ND	0.897 ± 0.055 ^c^	ND	0.423 ± 0.146 ^b^
16	Benzene, 1,2,4,5-tetramethyl-	C_10_H_14_	95-93-2	ND	ND	9.323 ± 0.280 ^b^	ND	ND
17	Benzene, 1-ethyl-2,4-dimethyl-	C_10_H_14_	874-41-9	ND	ND	3.700 ± 0.252 ^b^	ND	4.137 ± 0.623 ^b^
18	Benzene, pentamethyl-	C_11_H_16_	700-12-9	ND	ND	3.547 ± 0.407 ^c^	ND	1.130 ± 0.308 ^b^
19	Benzene, 1,2,3,5-tetramethyl-	C_10_H_14_	527-53-7	ND	ND	8.500 ± 0.440 ^b^	ND	ND
20	Benzene, 2-ethyl-1,4-dimethyl-	C_10_H_14_	1758-88-9	ND	ND	2.430 ± 0.234 ^b^	ND	0.577 ± 0.096 ^c^
21	Benzene, 1-methyl-4-propyl-	C_10_H_14_	1074-55-1	ND	ND	0.457 ± 0.012 ^b^	ND	ND
22	2,4-Di-tert-butylphenol	C_14_H_22_O	96-76-4	ND	ND	ND	ND	0.590 ± 0.020 ^b^
23	Benzene, 1-ethyl-3,5-dimethyl-	C_10_H_14_	934-74-7	ND	ND	ND	ND	0.920 ± 0.485 ^b^
24	2-Propenal	C_3_H_4_O	107-02-8	ND	ND	ND	0.600 ± 0.044	ND
25	Benzaldehyde, 3,4-dimethyl-	C_9_H_10_O	5973-71-7	ND	ND	2.957 ± 2.561 ^b^	ND	5.510 ± 0.907 ^c^
26	Decanal	C_10_H_20_O	112-31-2	ND	ND	0.783 ± 0.035 ^b^	ND	ND
27	Nonanal	C_9_H_18_O	124-19-6	9.957 ± 3.669 ^b^	1.857 ± 0.136 ^a^	1.133 ± 0.162 ^a^	1.817 ± 1.436 ^a^	0.793 ± 0.121 ^a^
28	Hydrogen isocyanate	CHNO	75-13-8	ND	0.531 ± 0.036 ^b^	5.0827 ± 0.173 ^c^	ND	ND
29	Formic acid, octyl ester	C_9_H_18_O_2_	112-32-3	ND	1.170 ± 0.363 ^b^	ND	ND	ND
30	Methyl formate	C_2_H_4_O_2_	107-31-3	ND	ND	ND	0.050 ± ND ^a^	1.173 ± 0.197 ^b^
31	Oxime-, methoxy-phenyl-_	C_8_H_9_NO_2_	1000222-86-6	26.417 ± 2.709 ^c^	31.740 ± 1.834 ^d^	8.430 ± 0.559 ^a^	34.863 ± 0.953 ^d^	18.093 ± 4.510 ^b^
32	Dodecane, 4,6-dimethyl-	C_14_H_30_	61141-72-8	ND	0.203 ± 0.077 ^b^	ND	0.271 ± 0.061 ^b^	ND
33	Nonane, 4,5-dimethyl-	C_11_H_24_	17302-23-7	ND	0.107 ± 0.035 ^b^	ND	ND	0.153 ± 0.021 ^c^
34	Naphthalene, 1,2,3,5,6,7,8,8a-octahydro-1,8a-dimethyl-7-(1-methylethenyl)-, [1R-(1.alpha.,7.beta.,8a.alpha.)]-	C_15_H_24_	4630-7-3	ND	0.773 ± 0.068 ^b^	ND	ND	ND
35	Benzene, 1-ethyl-4-(1-methylethyl)-	C_11_H_16_	4218-48-8	ND	ND	0.623 ± 0.055 ^b^	ND	ND
36	Benzene, 1-ethyl-2,4,5-trimethyl-	C_11_H_16_	17851-27-3	ND	ND	ND	ND	0.587 ± 0.032 ^b^
37	2-Oxo-4-phenyl-6-(4-chlorophenyl)-1,2-dihydropyrimidine	C_16_H_11_ClN_2_O	24030-13-5	ND	ND	ND	0.363 ± 0.015 ^b^	ND
38	3,5-Dimethylbenzylhexylamine	C_15_H_25_N	1000491-60-9	ND	ND	0.090 ± 0.082 ^b^	ND	ND
39	Cyclobutanecarbohydrazide	C_5_H_10_N_2_O	1000489-21-7	ND	ND	0.140 ± 0.030 ^b^	ND	ND
40	1,2-Benzenediol, O-(4-ethylbenzoyl)-O’-propargyloxycarbonyl-	C_19_H_16_O_5_	1000329-75-1	ND	ND	0.153 ± 0.023 ^b^	ND	ND
41	2-Butanol, 3-methyl-	C_5_H_12_O	598-75-4	ND	ND	ND	0.473 ± 0.410 ^b^	ND

ND: not detected. ^a–d^ Different letters in peer data indicate significant differences between groups (*p* < 0.05).

## Data Availability

The datasets used and analyzed during the current study are available from the corresponding author on reasonable request.
